# Formation of a calcium oxalate urethral stone in a 3‐year‐old boy due to hypocitraturia

**DOI:** 10.1002/iju5.12140

**Published:** 2020-01-13

**Authors:** Nobuhisa Kita, Yoshiro Nagao, Yoshiyuki Nabeshima, Ichiro Yamane, Masaaki Hirata, Kuniya Hatakeyama

**Affiliations:** ^1^ Department of Pediatrics Fukuoka Tokushukai Hospital Kasuga Fukuoka Japan; ^2^ Department of Urology Fukuoka Tokushukai Hospital Kasuga Fukuoka Japan

**Keywords:** calcium oxalate, citrates, pediatrics, urinary calculi, urolithiasis

## Abstract

**Introduction:**

Urolithiasis in children is often due to metabolic abnormalities (e.g. hypocitraturia) and hence recurs frequently.

**Case presentation:**

A 3‐year‐old boy presented with gross hematuria. Computed tomography detected a urethral calculus. The calculus was removed surgically. The stone was composed of calcium oxalate. Although oxalate and uric acid levels in the urine were within normal ranges, urine calcium was moderately elevated and urine citrate was substantially low. Urinalyses of the parents revealed that the father had acidic hypocitraturic urine, containing oxalate crystals, and the mother had hypercalciuria. Administration of oral citrate acid normalized urine citrate levels and eliminated the oxalate crystals, from the boy and his father.

**Conclusion:**

Although preventing urolithiasis using oral citrate is common in the adult population, this preventive measure is not well recognized in children, thus warranting further study.

**Keynote message**

A 3‐year‐old boy developed a urethral stone composed of calcium oxalate, possibly due to hypocitraturia. The patient's father was also hypocitraturic. Oral citrate was effective in preventing urolithiasis in both the boy and his father. As the westernization in food preferences in Japan and other Asian countries may increase pediatric urolithiasis, effectiveness of this preventive measure for children warrant further studies.

## Introduction

Urinary stones in children are rare but recur frequently because metabolic abnormalities often underlie pediatric urolithiasis.^1^, ^2^ We report a pediatric case of a urethral stone. The stone was composed of calcium oxalate and caused by hypocitraturia.

## Case presentation

**Figure 1:**
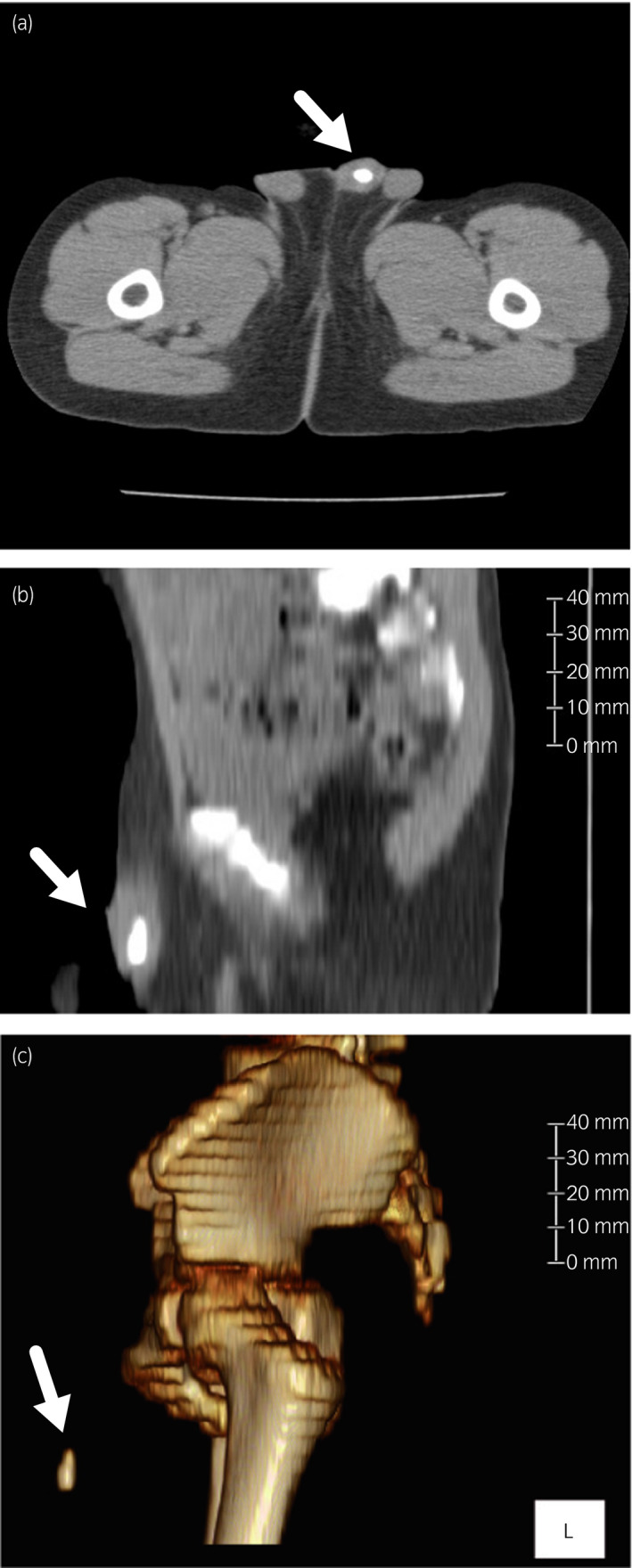
(a) Computed tomography of the urethral stone in the patient. The transverse view reveals a urethral stone (8 × 8 mm). (b) The length of the stone was 1 cm in the sagittal view. (c) The three‐dimensional reconstruction shows a droplet‐shaped stone.

A 3‐year‐old Japanese boy was diagnosed with microhematuria during a regular urinary test. He was otherwise healthy, with normal growth (14.9 kg, 99 cm), and without any remarkable past medical history or family history. He had not developed urinary tract infection previously. He was referred to a nearby hospital, where ultrasonography revealed a 6‐mm‐diameter stone in his bladder. Three months later, he presented to our hospital with penile pain. He did not have hypospadiasis. A computed tomography scan revealed a stone (1 cm in length; cross‐section of 8 × 8 mm) impacting the urethra (Fig. 1).

Blood analyses detected no abnormalities. Serum creatinine (27 μmol/L, or 0.31 mg/dL in conventional units), C‐reactive protein (1.9 nmol/L, 0.02 mg/dL), serum calcium (2.4 mmol/L, 9.8 mg/dL), magnesium (0.82 mmol/L, 2.0 mg/dL), phosphorus (1.5 mmol/L, 4.6 mg/dL), intact parathyroid hormone (2.5 pmol/L, 24 pg/mL), 1,25‐(OH)_2_ vitamin D (151 pmol/L, 63 pg/mL), 25‐OH vitamin D (67 nmol/L, 27 ng/mL), aldosterone (0.29 nmol/L, 104 pg/mL), and renin activity (1.3 ng/mL/h) were all within the normal ranges. The venous blood gas analysis showed that acidity (pH 7.368), bicarbonate (23 mmol/L), anion gap (10 mmol/L), base excess (−1.7 mmol/L), and ionized calcium (1.27 mmol/L) were within the reference ranges.

However, his urine was acidic (pH 5.5), contained noticeable numbers of red blood cells (30–49/high power field) and white cells (10–19/high power field). A urine culture did not yield bacteria.

**Figure 2:**
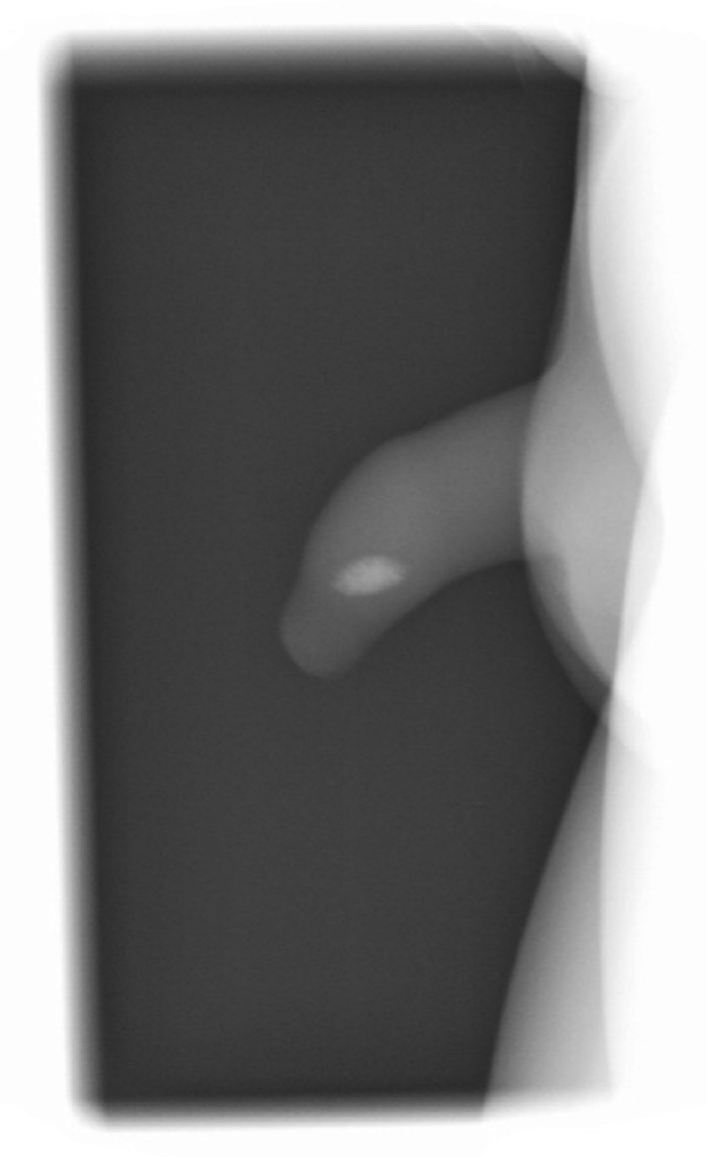
Plain X‐ray of the urethra. The calculus was in the navicular fossa of urethra, and was very close to the orifice.

Physical removal of the stone was attempted the next day. Initially, we prepared for endoscopic lithotripsy. However, the X‐ray showed that the stone was located close to the orifice (Fig 2). Therefore, under general anesthesia, a mosquito clamp was inserted gently into the outer opening of the urethra. The stone was visible. The calculus was fractured carefully by the mosquito clamp and removed. Ultrasonography and X‐ray fluoroscopy did not detect a remaining fragment. The hematuria diminished, and he was discharged. The stone was composed of 98% calcium oxalate.

Subsequently, the boy was readmitted to the hospital for a 24‐hour urine storage test (Table 1). He urinated normal levels of oxalate and uric acid, but slightly elevated calcium. Remarkably, the citrate level was substantially low. The urine was acidic (pH 5.5) and contained oxalate crystals (Table 2). Therefore, we administered oral potassium/sodium citrate (Uralyt‐U^®^; Nippon Chemiphar, Tokyo, Japan) at 1 g/day, to prevent recurrent urolithiasis.^3^ The urine citrate and calcium normalized, and the calcium oxalate crystals diminished (Table 2). The urinary excretion of calcium also decreased.^4^

**Table 1:** Results of the 24‐hour urine storage test

Parameter	Patient level	Normal range
Oxalate	42 mg/1.73 m^2^/day	<60 mg/1.73 m^2^/day
Uric acid	17 μmol/L GFR† 0.28 mg/dL GFR†	<33 μmol/L GFR† <0.56 mg/dL GFR†
Calcium (/kg/day)	160 μmol/kg/day 6.4 mg/kg/day	<100 μmol/kg/day <4 mg/kg/day
Calcium (/g creatinine)	6.7 mmol/g creatinine 0.27 g/g creatinine	<5.2 mmol/g creatinine <0.21 g/g creatinine
Citrate	196 mg/g creatinine	>400 mg/g creatinine

We showed the numerical results both in International System of Units and in conventional units, thereafter, to ease understanding.

† GFR, glomerular filtration rate. Uric acid/glomerular filtration rate was approximated by the equation: urine uric acid/urine creatinine × plasma creatinine.

**Table 2:** Urinalyses results of family members

Parameter	Patient (before oral citrate)†	Patient (with oral citrate)‡	Father (before oral citrate)‡	Father (with oral citrate)‡	Mother‡
pH	5.5	7.0	5.5	7.0	6.5
Citrate (mg/g creatinine) (normal value: >400)	196	553	272	687	507
Calcium oxalate crystals	+	−	+	−	−
Calcium (mmol/g creatinine) (normal value: <5.2)	6.7	4.5	2.7	2.1	7.7
Calcium (g/g creatinine) (normal value: <0.21)	0.27	0.18	0.11	0.083	0.31

†24‐hour storage; ‡Tests were performed in two or more time points and averaged.

We also tested the parents of our patient. The results of blood tests were unremarkable in both parents. Urine calcium level was slightly elevated in the mother (7.7 mmol/g creatinine or 0.31 g/g creatinine). Remarkably, the father's urine was acidic (pH 5.5), had a low level of citrate, and contained calcium oxalate crystals (Table 2). An ultrasonography did not detect any urinary stones. To prevent stone formation, the father started oral potassium/sodium citrate at 2 g/day. The urinary acidity and citrate normalized, and oxalate crystals disappeared (Table 2). We did not treat the mother.

We conducted computed tomography urography of the boy, 9 months after the stone removal. No anatomical abnormality was identified (Fig. S1). Voiding cystourethrography did not detect reflex (data not shown).

We have been monitoring urine of the boy and his father every 3 months, focusing upon acidity, citrate, hematuria, and oxalate crystals. Ultrasonography of the two has been conducted every half a year. To date, neither the boy nor his father developed any new stones, crystals, hematuria, or urinary tract infection.

Written informed consent was obtained from the father of the boy. The institutional review board of our hospital approved the publication of this report (G1905).

## Discussion

Calcium oxalate stone is common.^3^ Restriction of calcium intake increases the risk of urinary stone.^5^, ^6^ Instead, oral citrate decreases urine acidity, thereby increasing the solubility of stones. Furthermore, citrate chelates calcium and hampers its binding to other molecules (e.g. oxalate). In addition, citrate decreases excretion of calcium^7^ because citrate‐induced systemic alkalinization decreases bone resorption and increases tubular calcium reabsorption.^4^

Increased consumption of protein, cholesterol, and purine in the meat may play a role in the surging prevalence of urolithiasis.^6^, ^8^, ^9^ Although our patient and his father had ordinary food preferences, they possibly consumed more meat than in Japanese food tradition which seldom used meat. However, restricting meat intake could have undesirable effects on childhood growth.

Instead, citrate does not have adverse effects if administered in an adequate dose. Oral citrate is a widely recognized prevention against urinary stones in adults in many countries.^8^, ^10^ Oral citrate is also recommended for children in the USA.^3^ However, little attention has been paid to medical prevention of urinary stones in Japanese children. As food choices in Japan and other Asian countries are rapidly being westernized, meat consumption is increasing. Therefore, medical prevention of urolithiasis is becoming increasingly important.

The identification of hypocitraturia in our 3‐year‐old patient led to the recognition of this condition in his father. Heredity plays a substantial role in calcium stones^9^, ^11^ and hypocitraturia.^12^ Preventive measures were effective in the child and his father. When a urinary stone is detected in a child, the presence of its causative factor should be explored in the family.

## Conclusion

A calcium oxalate urethral stone was removed from a 3‐year‐old boy. The causative factor, hypocitraturia, was shared by his father. We initiated oral citrate in both the patient and his father. Identifying the causative factor and preventing formation of new stones are important for both the patient and his/her family.

## Conflict of interest

The authors declare no conflict of interest.

## Supporting information


**Figure S1.** Absence of anatomical abnormality in the urinary tract of the boy. Computed tomography urography did not identify a new stone, anatomical abnormalities, or a ureteral dilation which would indicate presence of a stricture (a). The lower half of the left ureter was truncated due to a normal peristaltic movement in the computed tomography urography. However, the plain X‐ray taken immediately after the computed tomography urography showed the intact ureters (arrows) (b).
